# Supplementary material for: Machine Learning Accelerates Crystallization for Structure Determination
Source: Angew Chem Int Ed Engl. 2026 May 4;65(25):e1218503. doi: 10.1002/anie.1218503 (PMC13266958; doi:10.1002/anie.1218503)

checkCIF/PLATON report

Structure factors have been supplied for datablock(s) lcz094

THIS REPORT IS FOR GUIDANCE ONLY. IF USED AS PART OF A REVIEW PROCEDURE FOR PUBLICATION, IT SHOULD NOT REPLACE THE EXPERTISE OF AN EXPERIENCED CRYSTALLOGRAPHIC REFEREE.

No syntax errors found.   CIF dictionary   Interpreting this report

Datablock: lcz094

```
Bond precision:   C-C = 0.0065 A          Wavelength=1.54184

Cell:            a=13.1122(2)      b=17.9462(3)      c=22.7626(3)
                 alpha=70.784(1)   beta=79.312(1)    gamma=75.974(2)
Temperature:     100 K
```

	Calculated	Reported
Volume	4874.37(14)	4874.37(14)
Space group	P -1	P -1
Hall group	-P 1	-P 1
Moiety formula	C41 H31 Ag3 F18 N10 O6 S2, 2(C15 H3 Ag3 F18 N6)	3(C15 H3 Ag3 F18 N6), 2(C13 H14 N2 O3 S)
Sum formula	C71 H37 Ag9 F54 N22 O6 S2	C71 H37 Ag9 F54 N22 O6 S2
Mr	3355.18	3355.17
Dx,g cm-3	2.286	2.286
Z	2	2
Mu (mm-1)	16.112	16.112
F000	3212.0	3212.0
F000'	3228.80	
h,k,lmax	16,22,28	16,22,28
Nref	19497	18309
Tmin,Tmax	0.045,0.123	0.325,1.000
Tmin'	0.007	

```
Correction method= # Reported T Limits: Tmin=0.325 Tmax=1.000
AbsCorr = MULTI-SCAN
```

Data completeness= 0.939 Theta(max)= 73.012

R(reflections)= 0.0346(17000) wR2(reflections)= 0.0923(18309)

S = 1.044 Npar= 1485

The following ALERTS were generated. Each ALERT has the format
test-name_ALERT_alert-type_alert-level.
Click on the hyperlinks for more details of the test.

Alert level C

```
PLAT029_ALERT_3_C _diffrn_measured_fraction_theta_full value Low .      0.970 Why?
PLAT042_ALERT_1_C Calc. and Reported MoietyFormula Strings  Differ     Please Check
              Calc: C41 H31 Ag3 F18 N10 O6 S2, 2(C15 H3 Ag3 F18 N6
              Rep.: 3(C15 H3 Ag3 F18 N6), 2(C13 H14 N2 O3 S)
PLAT213_ALERT_2_C Atom F03J            has ADP max/min Ratio .....        3.8 prolat
PLAT213_ALERT_2_C Atom F04L            has ADP max/min Ratio .....        3.5 prolat
PLAT214_ALERT_2_C Atom F041      (Anion/Solvent) ADP max/min Ratio        4.2 prolat
PLAT220_ALERT_2_C NonSolvent   Resd 1  F   Ueq(max)/Ueq(min) Range        3.8 Ratio
PLAT420_ALERT_2_C D-H Bond Without Acceptor  N027     --H027     .     Please Check
PLAT911_ALERT_3_C Missing FCF Refl Between Thmin & STh/L=    0.600        524 Report
               13  3  0,  15  3  0,  15  4  0,  13  5  0,  15  5  0,  13  6  0,
               14  6  0,  15  6  0,  14  7  0,  15  7  0,  13  8  0,  14  8  0,
               15  8  0,  13  9  0,  14  9  0,  12 10  0,  13 10  0,  14 10  0,
               13 11  0,  14 11  0,  13 12  0,  14 12  0,  13 13  0,  12 14  0,
               13 14  0,  12 15  0, -12-15  1, -12-14  1, -13-13  1, -12-13  1,
                   (  494 More Missing: see the .ckf listing file)
PLAT971_ALERT_2_C Check Calcd Resid. Dens.  0.94Ang From F04L          1.56 eA-3
PLAT972_ALERT_2_C Check Calcd Resid. Dens.  0.84Ang From Ag08         -1.80 eA-3
PLAT972_ALERT_2_C Check Calcd Resid. Dens.  0.81Ang From Ag03         -1.75 eA-3
PLAT972_ALERT_2_C Check Calcd Resid. Dens.  0.81Ang From Ag04         -1.73 eA-3
PLAT972_ALERT_2_C Check Calcd Resid. Dens.  0.94Ang From Ag01         -1.63 eA-3
PLAT972_ALERT_2_C Check Calcd Resid. Dens.  0.78Ang From Ag05         -1.63 eA-3
PLAT972_ALERT_2_C Check Calcd Resid. Dens.  0.85Ang From Ag02         -1.61 eA-3
PLAT972_ALERT_2_C Check Calcd Resid. Dens.  0.79Ang From Ag07         -1.61 eA-3
```

Alert level G

```
PLAT003_ALERT_2_G Number of Uiso or U(i,j)  Restrained non-H-Atoms          1 Report
PLAT007_ALERT_5_G Number of Unrefined Donor-H Atoms .............          6 Report
              H00J  H00U  H015  H01T  H027  H028
PLAT083_ALERT_2_G SHELXL Second Parameter in WGHT  Unusually Large      13.86 Why ?
PLAT186_ALERT_4_G The CIF-Embedded .res File Contains ISOR Records          1 Report
PLAT242_ALERT_2_G Low    'MainMol' Ueq as Compared to Neighbors of       C032 Check
PLAT242_ALERT_2_G Low    'MainMol' Ueq as Compared to Neighbors of       C033 Check
PLAT242_ALERT_2_G Low    'MainMol' Ueq as Compared to Neighbors of       C03M Check
PLAT242_ALERT_2_G Low    'MainMol' Ueq as Compared to Neighbors of       C03P Check
PLAT242_ALERT_2_G Low    'MainMol' Ueq as Compared to Neighbors of       C043 Check
PLAT242_ALERT_2_G Low    'MainMol' Ueq as Compared to Neighbors of       C04C Check
PLAT242_ALERT_2_G Low    'MainMol' Ueq as Compared to Neighbors of       C038 Check
PLAT242_ALERT_2_G Low    'MainMol' Ueq as Compared to Neighbors of       C03G Check
PLAT242_ALERT_2_G Low    'MainMol' Ueq as Compared to Neighbors of       C048 Check
PLAT242_ALERT_2_G Low    'MainMol' Ueq as Compared to Neighbors of       C04B Check
```

```
PLAT242_ALERT_2_G Low    'MainMol' Ueq as Compared to Neighbors of       C04D Check
PLAT242_ALERT_2_G Low    'MainMol' Ueq as Compared to Neighbors of       C04F Check
PLAT242_ALERT_2_G Low    'MainMol' Ueq as Compared to Neighbors of       C03R Check
PLAT242_ALERT_2_G Low    'MainMol' Ueq as Compared to Neighbors of       C03S Check
PLAT242_ALERT_2_G Low    'MainMol' Ueq as Compared to Neighbors of       C03T Check
PLAT242_ALERT_2_G Low    'MainMol' Ueq as Compared to Neighbors of       C03U Check
PLAT242_ALERT_2_G Low    'MainMol' Ueq as Compared to Neighbors of       C03Y Check
PLAT242_ALERT_2_G Low    'MainMol' Ueq as Compared to Neighbors of       C04E Check
PLAT434_ALERT_2_G Short Inter HL..HL Contact F019     ..F02C     .       2.69 Ang.
                                                  x,y,z  =      1_555 Check
PLAT720_ALERT_4_G Number of Unusual/Non-Standard Labels ..........        201 Note
              Ag01    Ag02    Ag03    Ag04    Ag05    Ag06    Ag07    Ag08
              Ag09    S00A    S00B    F00C    F00D    F00E    F00F    F00G
              F00H    F00I    O00J    H00J    F00K    F00L    F00M    F00N
              F00O    F00P    F00Q    F00R    F00S    F00T    O00U    H00U
              F00V    F00W    F00X    F00Y    F00Z    F010    F011    O012
              F013    F014    O015    H015    F016    F017    N018    F019
              F01A    N01B    N01C    F01D    F01E    N01F    O01G    F01H
              F01I    F01J    N01K    N01L    N01M    F01N    N01O    N01P
              F01Q    F01R    N01S    O01T    H01T    F01U    N01V    N01W
              N01X    N01Y    N01Z    N020    N021    N022    F023    F024
              F025    N026    N027    H027    N028    H028    F029    F02A
              F02B    F02C    F02D    N02E    F02F    F02G    C02H    F02I
              C02J    C02K    C02L    C02M    C02N    H02N    C02O    C02P
              C02Q    C02R    C02S    H02S    C02T    C02U    C02V    C02W
              C02X    H02X    F02Y    C02Z    C030    H030    C031    H031
              C032    C033    C034    C035    C036    H036    C037    C038
              C039    C03A    H03A    C03B    H03B    C03C    C03D    C03E
              C03F    C03G    C03H    H03H    C03I    H03I    F03J    C03K
              H03K    C03L    H03L    C03M    C03N    H03N    C03O    C03P
              C03Q    C03R    C03S    C03T    C03U    C03V    C03W    C03X
              H03X    C03Y    C03Z    C040    F041    C042    H042    C043
              C044    C045    C046    H046    C047    H047    C048    C04A
              H04G    H04J    H04K    C04B    C04C    C04D    C04E    C04F
              C04G    H04L    H04M    H04N    C04H    H04H    C04I    H04I
              C04J    H04A    H04B    H04C    C04K    H04D    H04E    H04F
              F04L
PLAT860_ALERT_3_G Number of Least-Squares Restraints ............          6 Note
PLAT883_ALERT_1_G Absent Datum for _atom_sites_solution_primary ..     Please Do !
PLAT910_ALERT_3_G Missing FCF Reflection(s) Below Theta(Min)[Deg]=       3.50 Note
                0  1  0,   0  0  1,   0  1  1,
PLAT912_ALERT_4_G Missing # of FCF Reflections Above STh/L=  0.600        659 Note
PLAT941_ALERT_3_G Average HKL Measurement Multiplicity ...........        4.6 Low
PLAT969_ALERT_5_G The 'Henn et al.' R-Factor-gap value ...........      4.326 Note
              Predicted wR2: Based on SigI**2  2.13 or SHELX Weight  8.85
PLAT978_ALERT_2_G Number C-C Bonds with Positive Residual Density.          0 Info
```

---

0 **ALERT level A** = Most likely a serious problem - resolve or explain
0 **ALERT level B** = A potentially serious problem, consider carefully
16 **ALERT level C** = Check. Ensure it is not caused by an omission or oversight
31 **ALERT level G** = General information/check it is not something unexpected

2 ALERT type 1 CIF construction/syntax error, inconsistent or missing data

```
 35 ALERT type 2 Indicator that the structure model may be wrong or deficient
  5 ALERT type 3 Indicator that the structure quality may be low
  3 ALERT type 4 Improvement, methodology, query or suggestion
  2 ALERT type 5 Informative message, check
```

Validation response form

Please find below a validation response form (VRF) that can be filled in and pasted into your CIF.

```
# start Validation Reply Form
_vrf_PLAT029_lcz094
;
PROBLEM: _diffrn_measured_fraction_theta_full value Low .      0.970 Why?
RESPONSE: ...
;
_vrf_PLAT042_lcz094
;
PROBLEM: Calc. and Reported MoietyFormula Strings  Differ     Please Check
RESPONSE: ...
;
_vrf_PLAT213_lcz094
;
PROBLEM: Atom F03J            has ADP max/min Ratio .....        3.8 prolat
RESPONSE: ...
;
_vrf_PLAT214_lcz094
;
PROBLEM: Atom F041      (Anion/Solvent) ADP max/min Ratio        4.2 prolat
RESPONSE: ...
;
_vrf_PLAT220_lcz094
;
PROBLEM: NonSolvent   Resd 1  F   Ueq(max)/Ueq(min) Range        3.8 Ratio
RESPONSE: ...
;
_vrf_PLAT420_lcz094
;
PROBLEM: D-H Bond Without Acceptor  N027     --H027     .     Please Check
RESPONSE: ...
;
_vrf_PLAT911_lcz094
;
PROBLEM: Missing FCF Refl Between Thmin & STh/L=    0.600        524 Report
RESPONSE: ...
;
_vrf_PLAT971_lcz094
;
PROBLEM: Check Calcd Resid. Dens.  0.94Ang From F04L           1.56 eA-3
RESPONSE: ...
;
_vrf_PLAT972_lcz094
;
```

```
PROBLEM: Check Calcd Resid. Dens.  0.84Ang From Ag08          -1.80 eA-3
RESPONSE: ...
;
# end Validation Reply Form
```

It is advisable to attempt to resolve as many as possible of the alerts in all categories. Often the minor alerts point to easily fixed oversights, errors and omissions in your CIF or refinement strategy, so attention to these fine details can be worthwhile. It is up to the individual to critically assess their own results and, if necessary, seek expert advice.

PLATON version of 17/09/2025; check.def file version of 17/09/2025

---

duplicate check

No duplication found

Datablock lcz094 - ellipsoid plot

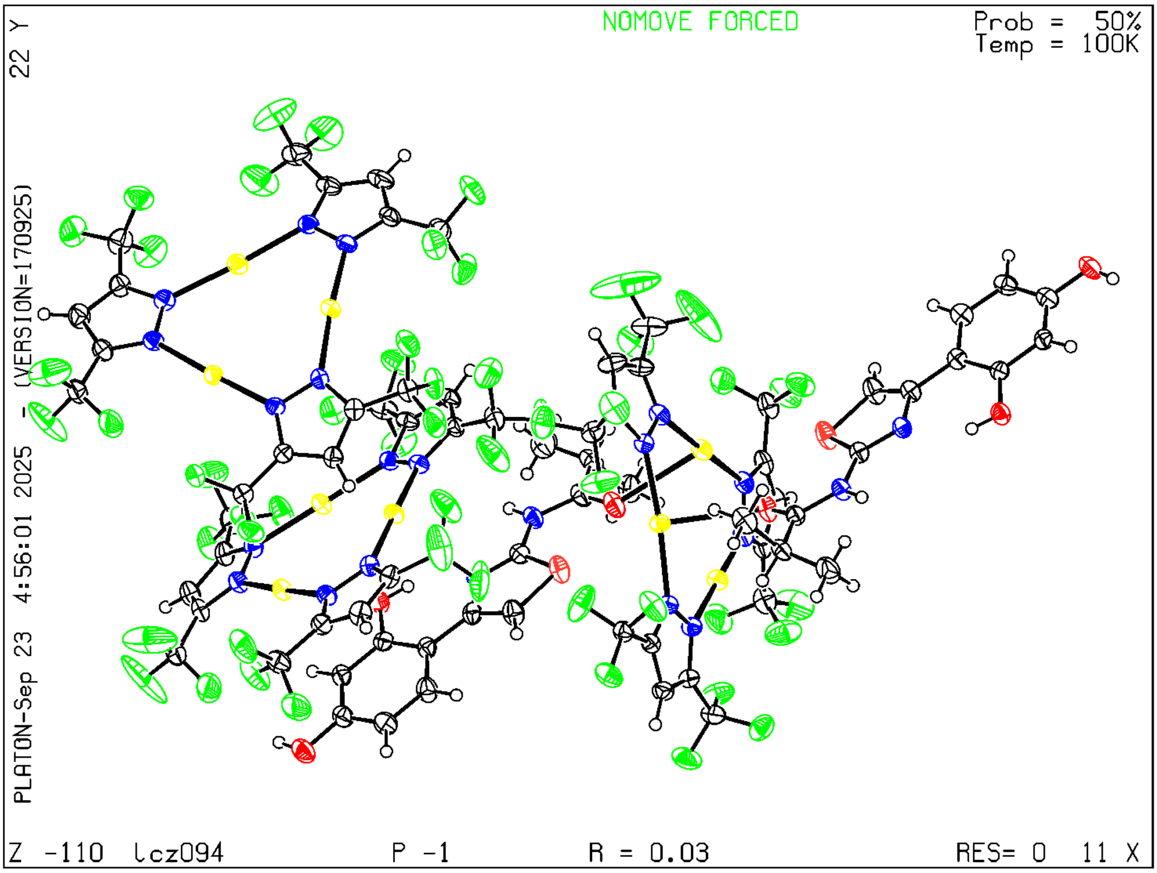

Supplement: Supplementary file 2 — Supporting File 2: anie72462‐sup‐0002‐DataFile.zip. [file ANIE-65-e1218503-s002.zip › supplementary_materials_crystallographic_data submit.zip!/cif_and_checkcif(1-114)/Ag3Pz3_94/Ag3Pz3_94_checkcif.pdf]
